# Sensitivity and specificity of lung cancer screening using chest low-dose computed tomography

**DOI:** 10.1038/sj.bjc.6604351

**Published:** 2008-05-06

**Authors:** Y Toyoda, T Nakayama, Y Kusunoki, H Iso, T Suzuki

**Affiliations:** 1 Department of Cancer Control and Statistics, Osaka Medical Center for Cancer and Cardiovascular Diseases, Osaka, Japan; 2 Public Health, Department of Social and Environmental Medicine, Graduate School of Medicine, Osaka University, Osaka, Japan; 3 Osaka Prefectual Medical Center for Respiratory and Allergic Diseases, Osaka, Japan

**Keywords:** sensitivity, specificity, lung cancer, screening

## Abstract

Lung cancer screening programmes using chest X-ray and sputum cytology are routinely performed in Japan; however, the efficacy is insufficient. Screening using low-dose computed tomography (CT) is a more effective approach and has the potential to detect the disease more accurately. A total of 7183 low-dose CT screening tests for 4689 participants and 36 085 chest X-ray screening tests for 13 381 participants were conducted between August 1998 and May 2002. Sensitivity and specificity of lung cancer screening were calculated by both the detection method and the incidence method by linkage of the screening database and the Cancer Registry database. The preclinical detectable phase was assumed to be 1 year. Sensitivity and specificity by the detection method were 88.9 and 92.6% for low-dose CT and 78.3 and 97.0% for chest X-ray, respectively. Sensitivity of low-dose CT by the incidence method was 79.5%, whereas that of chest X-ray was 86.5%. Lung cancer screening using low-dose CT resulted in higher sensitivity and lower specificity than traditional screening according to the detection method. However, sensitivity by the incidence method was not as high as this. These findings demonstrate the potential for overdiagnosis in CT screening-detected cases.

Lung cancer is the leading cause of cancer death in Japan, with 45 927 men and 17 307 women dying from lung cancer in 2006. Since 1987, lung cancer screening programme using chest X-ray and sputum cytology for all residents aged 40 years of age and older regardless of smoking status has been conducted by the Ministry of Health and Welfare. Unfortunately, the efficacy of lung cancer screening using chest X-ray and sputum cytology is insufficient (Fontana *et al*, 1986; Marcus *et al*, 2000a, 2006b; Sagawa *et al*, 2003a). Therefore, a more effective approach is required to decrease lung cancer deaths.

Annual lung cancer screening using low-dose computed tomography (CT) has been performed as an opportunistic screening method since the early 1990s in Japan. Several study groups introduced low-dose CT for population-based screening in clinical trials. These previous studies reported a high detection rate, an ability to detect small tumours and a high survival rate in detected cases (Henschke *et al*, 2001, 2006; Sone *et al*, 2001; Nawa *et al*, 2002; Sobue *et al*, 2002a; Swensen *et al*, 2002; Diederich *et al*, 2004; Jett, 2005; Libby *et al*, 2006). Some studies referred to interval cancer cases of lung cancer screening using low-dose CT, and one study referred to the sensitivity of screening (Sone *et al*, 2001; Diederich *et al*, 2004). However, screening databases are yet to be linked to a cancer registry, which is essential for accurate evaluation of screening, including the confirmation of all interval cancer cases. To date, no study has been conducted on sensitivity and specificity of annual lung cancer screening using low-dose CT and cancer registry data. Therefore, the present study was conducted to evaluate sensitivity and specificity of annual lung cancer screening using low-dose CT and data from screening and local cancer registry databases.

## MATERIALS AND METHODS

### Study setting

Since 1998, annual population-based lung cancer screening using low-dose CT has been conducted at five municipalities in Osaka prefecture: A (city), B (city), C (town), D (town) and E (town). All residents aged 40 years of age and older were recruited by mail using a letter from the public health division of each municipality regardless of smoking status. Subjects recruited to the lung cancer screening programme underwent either miniature chest X-ray or low-dose chest CT.

As a principle, heavy smokers were recommended to undergo low-dose CT screening. In addition, the persons who want to undergo low-dose CT screening also underwent low-dose CT screening. Others underwent chest X-ray screening.

A high-risk group for lung cancer, smokers with over a 20 pack index or who had haemosputum, was examined by 3-day pooled sputum cytology.

Low-dose CT or chest X-ray images were reviewed and classified by two trained physicians to determine the need for further clinical examination. Sputum cytology was also performed by a certified cytopathologist to determine the need for further clinical examination.

Those diagnosed with the need for further clinical examination were regarded as screen-positive. These individuals were asked to undergo further diagnostic evaluation at Osaka Medical Center for Cancer and Cardiovascular Disease. All individuals with positive chest X-ray screening were asked to undergo chest CT as a further examination.

### Data collection

All subjects were individuals who had undergone either low-dose CT or chest X-ray screening tests between August 1998 and May 2002. The following participants were excluded from the analyses: (1) participants who had a past history of lung cancer, (2) participants who were suspected of having lung cancer by a previous screening or other medical examination and had received medical treatment and (3) participants who were suspected of having lung cancer at the previous screening or by other medical examination, but had refused further examinations.

Participants were divided into two groups: (a) low-dose CT group and (b) chest X-ray group. The low-dose CT group consisted of persons who had undergone low-dose CT at least one time during the study period, whereas the chest X-ray group consisted of persons who had undergone only chest X-ray. The low-dose CT group included those who had undergone both CT screening and chest X-ray screening within the study period. For these cases, screenings using chest X-ray were ignored to evaluate low-dose CT screening.

All data were entered into the screening database that was linked to the Osaka Cancer Registry (OCR) database with data reflecting incidence cases through December 2003. The indices used to collate the two databases were name, sex, address and date of birth. Information about lung cancer cases was extracted from hospital medical records or the OCR file.

We assumed that the preclinical detectable phase was 1 year for interval cancer cases. For death certificate-only cases, the date of 3 months before death was regarded as the date of diagnosis. Using these parameters, all lung cancer cases diagnosed within 1 year after a negative screen were regarded as interval lung cancers. Screen-detected cases were considered as true-positive cases regardless of the time between the date of screening and the date of diagnosis.

### Statistical analyses

The sensitivity of screening was calculated by both the detection method and the incidence method. Although the detection method is simple and widely used, sensitivity estimated by detection method is affected by length and overdiagnosis biases (Day, 1985). The incidence method is not affected by length or overdiagnosis bias and is often used for breast cancer screening or colorectal cancer screening (Fletcher *et al*, 1993; Zappa *et al*, 2001).

### Detection method

Sensitivity and specificity were calculated by the detection method using the following formulae. 
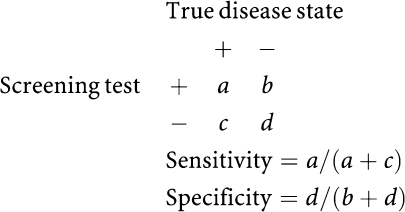


Sensitivity and specificity calculated by the detection method were stratified by smoking status, histological type and screening rank. The screening rank was classified as the initial and repeated screenings, regardless of the number of years since the initial screening.

### Incidence method

In addition, we calculated sensitivity by the incidence method using the following approximate formula (Day, 1985; Zappa *et al*, 2001): 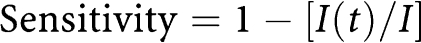


Where *I*(*t*)=the observed number of interval cancer cases during time *t* and *I*=the expected number of cases in the absence of screening.

We calculated the number of expected lung cancer cases in the absence of screening based on the following data. Age-specific lung cancer incidence rates provided from the OCR in 2001 were 16.3, 61.6, 180.9, 477.3 and 770.2 (per 100 000 person-years) for men, and 6.3, 25.9, 53.4, 116.7 and 241.3 for women, for age groups 40–49, 50–59, 60–69, 70–79 and ⩾80, respectively. Lung cancer incidence rates in the OCR were weighted by smoking status. According to the previous large-scale cohort study in Japan, the lung cancer incidence rates among ex-smokers and current smokers were assumed to be 2.2 times and 4.5 times of that of nonsmokers, respectively, among men, and 3.7 times and 4.2 times, respectively, among women (Sobue *et al*, 2002b). According to an official report from Osaka prefecture in 2003, the proportions of current smokers, ex-smokers and nonsmokers were 40, 30 and 30% among men, and 11, 7 and 82% among women, respectively (Department of public health, Osaka Prefecuture, 2006).

We assumed that smoking status proportions were the same across all age groups, so the expected incidence rate according to sex and smoking status was modified using the following formulae: 
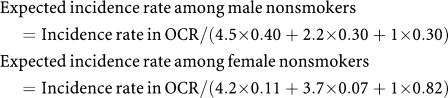


The expected incidence rates for ex-smokers and current smokers were assumed to be 2.2 times and 4.5 times of that of nonsmokers, respectively, among men, and 3.7 times and 4.2 times, respectively, among women.

The differences in sensitivity and specificity among the stratified variables were tested by *χ*^2^ test. All statistical analyses were performed using SAS software, version 8.01 (SAS Institute Inc., Cary, NC, USA).

### Ethical approval

The protocol for the present study was approved by the Ethics Committee of Osaka Medical Center for Cancer and Cardiovascular Disease, Osaka, Japan. Informed consent for participation in the clinical trial, including CT screening, was obtained from all individuals.

## RESULTS

From August 1998 to May 2002, a total of 7190 low-dose CT screening tests and a total of 36 085 chest X-ray screening tests were performed. Seven screening participants were excluded from analysis because they did not meet the eligibility criteria. Participants were ineligible for the following reasons: two participants were under follow-up care, one was suspected of having lung cancer but refused further examination and four had a history of lung cancer. A total of 7183 low-dose CT screening tests for 4689 participants (2765 men and 1924 women) and 36 085 chest X-ray screening tests for 13 381 participants (4180 men and 9201 women) enrolled in the study.

Table 1 shows the number of screening tests by sex, age group, smoking status and rank of screening tests. Most of the participants who underwent low-dose CT screening were male current smokers or ex-smokers. Sputum cytology was additionally performed for 3539 screening tests for the low-dose CT group and 5417 screening tests for the chest X-ray group.

Forty cases in the low-dose CT group and 29 cases in the chest X-ray group were detected by the screening. Five interval cases in the low-dose CT group and eight interval cases in the chest X-ray group were confirmed by linkage to OCR (Table 2). All of the interval cancer cases for both the low-dose CT group and the chest X-ray group were smokers. As for the low-dose CT group, all of them were nonadenocarcinoma. Two cases, one in the low-dose CT group and one in the chest X-ray group, were detected by sputum cytology on negative radiological screen.

Table 3 shows sensitivity and specificity by the detection method according to histological type, smoking status and rank of screening. As a result, sensitivity and specificity (95% confidence interval) of screening were 88.9% (79.7–98.1%) and 92.6% (92.0–93.2%) for the low-dose CT group, and 78.3% (65.1–91.6%) and 97.0% (96.9–97.2%) for the chest X-ray group, respectively. Specificity of chest X-ray screening was significantly higher than that of low-dose CT screening (*P*<0.001). The difference in sensitivity by the detection method was not significant.

As for histological type, sensitivity for adenocarcinoma was significantly higher than that for nonadenocarcinoma (low-dose CT: 100 *vs* 61.5%; *P*<0.001, and chest X-ray: 95.8 and 50.0%; *P*<0.001); however, the histological type of three interval cases in the chest X-ray group was unknown. As for screening rank, specificity for the repeated screenings was significantly higher than that for the initial screenings (low-dose CT: 95.7 *vs* 91.0%; *P*<0.001, and chest X-ray: 97.7 *vs* 95.9%; *P*<0.001). As for sex, specificity for men was significantly lower than that for women (low-dose CT: 92.1 *vs* 93.5%; *P*<0.05, and chest X-ray: 95.7 *vs* 97.6%; *P*<0.001). Sensitivity of chest X-ray screening for women was significantly higher than that for men (100 *vs* 68.2%; *P*<0.05). As for smoking status, sensitivity of both low-dose CT and chest X-ray for nonsmokers was 100%.

Table 4 shows sensitivity estimated by the incidence method. Until the end of December 2003, a total of 14 434 person-years (total for men: 9173 person-years; total for women: 5512 person-years) for the low-dose CT group and a total of 59 725 person-years (total for men: 17 962 person-years; total for women: 41 763 person-years) for the chest X-ray group had been followed up for. The mean follow-up terms were 3.1 person-years and 4.5 person-years, respectively. The number of expected lung cancer cases was calculated to be 24.4 persons for the low-dose CT group and 59.3 persons for the chest X-ray group. As a result, sensitivity (95% confidence interval) estimated by the incidence method was 79.5% (63.5–95.5%) and 86.5% (77.8–95.2%), respectively. The difference in sensitivity by the incidence method was not statistically significant.

## DISCUSSION

The present study is the first report on sensitivity and specificity of annual lung cancer screening using low-dose CT and data from a local Cancer Registry. Sensitivity and specificity of low-dose CT screening according to the detection method were 88.9 and 92.6%. The sensitivity estimated by the incidence method resulted in a value of 79.5%. On the other hand, sensitivity and specificity of chest X-ray in the same time frame by the detection method were 78.3 and 97.0%, respectively. Furthermore, sensitivity of chest X-ray screening by the incidence method was 86.5%.

In previous studies conducted in the1980s, sensitivity and specificity of annual lung cancer screening using chest X-ray and sputum cytology were also evaluated by the detection method. In those studies, sensitivity and specificity for usual screening were 63.6–88.0% and 94.7–99.6%, respectively (Sobue *et al*, 1991c; Soda *et al*, 1993; Sagawa *et al*, 1994b; Tsukada *et al*, 2002). The use of low-dose CT screening resulted in a higher sensitivity and lower specificity than usual screening. The reported high sensitivity in participants undergoing low-dose CT screening is the result of improvement in the detection of small tumours. The lower specificity value indicates the difficulty of diagnosing nodules detected by screening.

Several points must be considered when the present study results are compared with previous results. Since 1980s, lung cancer incidence by histological type has undergone a change over time. With a large decline in the smoking rate among men, the proportion of squamous cell carcinoma or small cell carcinoma has decreased, whereas the proportion of adenocarcinoma has increased (Yoshimi *et al*, 2003). The current environment may be more advantageous for lung cancer screening because adenocarcinoma occurring in the peripheral lung has a longer doubling time than squamous cell carcinoma (Arai *et al*, 1994). In addition, as most low-dose CT screening-detected lung cancer lesions are too small to detect by chest X-ray and have a longer preclinical phase, simple comparison of low-dose CT screening with chest X-ray screening is difficult.

We used the detection method and stratified analyses by screening rank and histological type. As for screening rank, specificity of both low-dose CT and chest X-ray for the repeated screenings was significantly higher than that of the initial screenings. The high specificity associated with repeated screenings is due to the fact that the review of previous images facilitates ruling out benign nodules. Sensitivity of low-dose CT and chest X-ray for the repeated screenings was lower than that of the initial screenings; however, the difference was not statistically significant. Sensitivity for the initial screenings was affected by length bias and overestimation because lung cancers with long preclinical detectable phases were more prevalent. Regarding histological type, adenocarcinoma sensitivity estimated by the detection method was significantly higher than that for nonadenocarcinoma for both low-dose CT and chest X-ray. In the previous study, sensitivity of chest X-ray was 86.4% for adenocarcinoma and 44.2% for nonadenocarcinoma (Sobue *et al*, 1991c). Both low-dose CT screening and chest X-ray screening have a high sensitivity for the detection of adenocarcinoma. In contrast, sensitivity estimated by the detection method for nonadenocarcinoma remained low. As for smoking status, both low-dose CT and chest X-ray had superior performance for nonsmokers.

Although the detection method is simple and widely used, it is affected by overdiagnosis or length bias because cancers with long preclinical detectable phases are included in the denominator. In the 1980s, lung cancer was considered to be an aggressive and rapid-growing cancer; however, it has been reported that low-dose CT screening-detected lung cancer has a long doubling time and good prognosis (Sone *et al*, 2001; Nawa *et al*, 2002; Sobue *et al*, 2002a; Swensen *et al*, 2002; Henschke *et al*, 2006; Libby *et al*, 2006). The incidence method, which is not affected by overdiagnosis bias and length bias, is preferred for the correct evaluation of low-dose CT screening. Screening for breast cancers or colorectal cancers, with long doubling times, has been evaluated using the incidence method whereas lung cancer screening has been evaluated using the detection method only (Fletcher *et al*, 1993; Zappa *et al*, 2001).

In this study, we calculated expected lung cancer incidence to be 24.4 persons for the low-dose CT group and 59.3 persons for the chest X-ray group according to age-specific lung cancer incidence rate in the OCR, smoking status in Osaka prefecture and the relative risk of lung cancer incidence associated with smoking according to a large-scale cohort study in Japan. Unexpectedly, the sensitivity of low-dose CT screening estimated by the incidence method (79.5%) was lower than that of chest X-ray screening (86.5%); however, the difference was not statistically significant. There are several possible explanations for this contradiction. First, the mean follow-up term of the low-dose CT group (3.1 person-years) was shorter than that of the chest X-ray group (4.5 person-years). Furthermore, the mean pack index of current smokers among the low-dose CT group (42 for men and 23 for women) was somewhat higher than that of the chest X-ray group (38 for men and 16 for women). Therefore, expected lung cancer incidence for the low-dose CT group might be underestimated. Second, four screen-detected cases among the chest X-ray group were checked with lesions other than cancer. These lung cancer cases were incidentally detected by the subsequent chest CT as a further examination on positive tests; all of them were adenocarcinoma. When these cases were regarded as interval cases, sensitivity (95% confidence interval) of chest X-ray screening by the incidence method resulted in 79.7% (69.5–90.0%). Considering these points, sensitivity of low-dose CT screening according to the incidence method with 3–5 person-years of follow-up period would be almost equal to that of chest X-ray screening. These findings suggest that the efficacy of low-dose CT screening might be limited to rapid-growing lung cancer with a short preclinical detectable phase (<=1 year). Since low-dose CT screening-detected lung cancer is slow growing, further research with a longer follow-up period is required.

A total of 40 lung cancer cases were detected by low-dose CT screening, suggesting the possibility of overdiagnosis by low-dose CT screening. In particular, low-dose CT screening detected 13 lung cancer cases in nonsmokers whereas expected incidence in nonsmokers was only 1.7 persons. All of these cases were peripheral adenocarcinoma. In contrast, expected lung cancer incidence for the chest X-ray group was higher than the number of screen-detected cases. This fact might suggest that there is little possibility of overdiagnosis by chest X-ray screening.

Of the five interval cancer cases in the low-dose CT group, four cases were squamous cell carcinoma or small cell carcinoma, which are strongly associated with smoking (Sobue *et al*, 1991d; Shimizu *et al*, 1994; Stellman *et al*, 2001). Three cases had remarkable emphysaema. These interval cancer cases associated with smoking indicate the limitation of low-dose CT screening for nonadenocarcinoma among smokers. In other words, the high sensitivity of low-dose CT screening identified using the detection method is due to the detection of adenocarcinoma with a long preclinical detectable phase.

This study has some limitations. First, many nodules were detected by low-dose CT screening, but subsequent pathological examinations were not performed. In this study, small pure ground-glass opacity nodules (<10 mm) were carefully observed, and no invasive treatment was performed. In these cases, lung cancer was highly suspected, but a lung cancer diagnosis was not made and the cases were not registered in the OCR. Given the presence of such cases, the sensitivity according to the detection method might be underestimated. Second, to compare usual screening with low-dose CT screening, the preclinical detectable phase was assumed to be 1 year. We need to assess a longer preclinical detectable phase, because most of the low-dose CT screening-detected lung cancer cases were slow growing. Third, the sample size was relatively small for proper evaluation, particularly for stratified analyses.

In summary, the present findings suggest that lung cancer screening using low-dose CT has a higher sensitivity and a lower specificity than usual lung cancer screening by chest X-ray, when using the detection method analysis. However, sensitivity estimated by the incidence method was not as high as that estimated by detection method. As all interval cancer cases were associated with smoking, low-dose CT screening showed limited efficacy for nonadenocarcinoma in smokers. Furthermore, these findings demonstrate the potential for overdiagnosis in low-dose CT screening-detected cases.

## Figures and Tables

**Table 1:** Number of screening tests performed by age group, smoking status and rank; (a) low-dose CT group and (b) chest X-ray group: Osaka, 1998–2002

	**Male**	**Female**	**Total**
*(a) Low-dose CT group*			
*Age (years)*			
40–49	700	490	1190
50–59	1147	1132	2279
60–69	1885	886	2771
70–79	690	194	884
80–	43	16	59
			
*Smoking status*			
Nonsmoker	362	2048	2410
Ex-smoker	1012	113	1125
Current smoker	3091	557	3648
			
*Rank*			
Initial	2765	1924	4689
Repeated	1700	794	2494
			
Total	4465	2718	7183
			
*(b) Chest X-ray group*			
*Age (years)*			
40–49	1258	4862	6120
50–59	1679	8632	10 311
60–69	4163	7910	12 073
70–79	2695	3670	6365
80–	573	643	1216
			
*Smoking status*			
Nonsmoker	2807	23 790	26 597
Ex-smoker	4328	740	5068
Current smoker	3233	1187	4420
			
*Rank*			
Initial	4180	9201	13 381
Repeated	6188	16 516	22 704
			
Total	10 368	25 717	36 085

CT=computed tomography.

**Table 2:** Interval cancer cases of screening; (a) low-dose CT group and (b) chest X-ray group

	**Sex**	**Age (years)**	**Pack index**	**Smoking status**	**Histological type**	**Location**	**Rank**	**Clinical stage**
*(a) Low-dose CT group*								
1	F	71	48	Current	Squamous	Unknown	Initial	III
2	M	60	43	Current	Large cell	Peripheral	Initial	III
3	M	72	48	Current	Small cell	Unknown	Repeated	IV
4	M	72	45	Current	Squamous	Unknown	Repeated	I
5^a^	F	59	29	Ex	Squamous	Central	Initial	I
								
*(b) Chest X-ray group*								
1	M	68	48	Current	Squamous	Unknown	Repeated	III
2	M	83	61	Current	Small cell	Unknown	Repeated	Unknown
3	M	72	21	Ex	Adeno	Unknown	Repeated	I
4	M	69	25	Current	Undifferentiated	Unknown	Initial	III
5	M	60	50	Current	Unknown	Unknown	Initial	Unknown
6^a^	M	63	68	Ex	Squamous	Central	Repeated	I
7	M	59	80	Current	Unknown	Unknown	Repeated	Unknown
8	M	85	15	Ex	Unknown	Unknown	Repeated	Unknown

CT=computed tomography.

a Detected by sputum cytology.

**Table 3:** Sensitivity and specificity by the detection method according to histological type, smoking status and rank of screening; (a) low-dose CT group and (b) chest X-ray group

	**No. of screenings**	**Screen-detected cases**	**Interval cases**	**Sensitivity (%) (95% CI)**	**Specificity (%) (95% CI)**
*(a) Low-dose CT group*					
*Sex*					
Men	4465	29	3	90.6 (80.5–100)	92.1 (91.3–92.9)
Women	2718	11	2	84.6 (65.0–100)	93.5 (92.6–94.4)
					
*Smoking status*					
Nonsmoker	2410	13	0	100	93.5 (92.5–94.4)
Ex-smoker	1125	6	1	85.7 (59.8–100)	91.5 (89.9–93.1)
Current smoker	3648	21	4	84.0 (69.6–98.4)	92.4 (91.6–93.3)
					
*Histological type*					
Adenocarcinoma	—	32	0	100	—
Nonadenocarcinoma	—	8	5	61.5 (35.1–88.0)	—
					
*Rank*					
Initial	4688	32	3	91.4 (82.2–100)	91.0 (90.2–91.8)
Repeated	2494	8	2	80.0 (55.2–100)	95.7 (94.9–96.5)
					
Total	7183	40	5	88.9 (79.7–98.1)	92.6 (92.0–93.2)
					
*(b) Chest X-ray group*					
*Sex*					
Men	10 368	15	8	65.2 (45.8–84.7)	95.7 (95.3–96.1)
Women	25 717	14	0	100	97.6 (97.3–97.8)
					
*Smoking status*					
Nonsmoker	26 597	13	0	100	97.4 (97.3–97.7)
Ex-smoker	5068	4	3	57.1 (20.5–93.8)	95.9 (95.3–96.4)
Current smoker	4420	12	5	70.6 (48.9–92.2)	95.7 (95.1–96.3)
					
*Histological type*					
Adenocarcinoma	—	23	1	95.8 (87.8–100)	—
Nonadenocarcinoma	—	6	4	50.0 (21.7–78.3)	—
Unknown	—	0	3	—	—
					
*Rank*					
Initial	13 381	13	2	86.7 (69.5–100)	95.9 (95.5–96.2)
Repeated	22 704	16	6	76.2 (58.0–94.4)	97.7 (97.5–97.9)
					
Total	36 085	29	8	78.3 (65.1–91.6)	97.0 (96.9–97.2)

CI=confidence interval; CT=computed tomography.

**Table 4:** Sensitivity of screening by the incidence method; (a) low-dose CT group and (b) chest X-ray group

	**Person-years**	**Expected incidence**	**Screen-detected cases**	**Interval cases**	**Sensitivity (%) (95% CI)**
*(a) Low-dose CT group*					
*Sex*					
Men	9173	21.8	29	3	86.2 (71.8–100)
Women	5512	2.5	11	2	20.0 (0–69.6)
					
* Smoking status*					
Nonsmokers	4878	1.7	13	0	100
Ex-smokers	2388	4.2	6	1	76.2 (35.5–100)
Current smokers	7419	18.6	21	4	78.5 (59.8–97.2)
					
Total	14 685	24.4	40	5	79.5 (63.5–95.5)
					
*(b) Chest X-ray group*					
*Sex*					
Men	17 962	42.1	15	8	81.0 (69.1–92.8)
Women	41 763	17.2	14	0	100
					
*Smoking status*					
Nonsmokers	42 976	17.4	13	0	100
Ex-smokers	8452	19.2	4	3	84.3 (68.1–100)
Current smokers	8297	22.8	12	5	78.1 (61.1–95.1)
					
Total	59 725	59.3	29	8	86.5 (77.8–95.2)

CI=confidence interval; CT=computed tomography.
